# Quantitation of estradiol by competitive light‐initiated chemiluminescent assay using estriol as competitive antigen

**DOI:** 10.1002/jcla.23014

**Published:** 2019-08-24

**Authors:** Jiuzhi Li, Liuxu Li, Ying Bian, Yang Yu, Zhonghua Qiang, Yuexiang Zhang, Huiqiang Li

**Affiliations:** ^1^ School of Medical Laboratory Tianjin Medical University Tianjin China; ^2^ Department of Clinical Laboratory Tianjin Central Hospital of Obstetrics and Gynecology Tianjin China

**Keywords:** equilibrium competitive assay, estradiol, estriol, light‐initiated chemiluminescent assay, quantitative analysis

## Abstract

**Background:**

Light‐initiated chemiluminescent assays (LICA) are homogeneous assays that are sensitive, specific, and free of separation and washing steps and have high throughput and high precision.

**Methods:**

In this research, we developed a competitive method by LICA to achieve accurate quantification of estradiol (E2) in human serum. E2 competed with estriol (E3) for binding to anti‐human E2 antibodies. E3 was linked to biotin via bovine serum albumin as a linker. As this assay used competition between the labeled tracer and the analyte, an increase in E2 concentration will cause a signal decrease.

**Results:**

The expected detection range of E2 was 20‐5000 pg/mL. The analytical and functional sensitivities were 7.16 and 13.7 pg/mL, respectively. The intra‐ and inter‐assay coefficients of variation were both below 15%, and the recovery rate ranged from 97.5% to 106.8%. The interference rates ranged from −3.6% to 5.4% and met detection requirements for E2 in hyperbilirubinemia, hemolysis, and lipemia in clinical samples. In addition, the cross‐reactivity rates between E2 and structural analogs and some reproductive hormones varied from 1.9% to 10.6% which showed that LICA is highly specific for E2. Moreover, our results showed high accordance with the IMMULITE 2000 (*y* = 0.6695*x* + 47.92, *r* ^2^ = .843) and VIDAS systems (*y* = 1.099*x* − 821.5, *r* ^2^ = .9392).

**Conclusion:**

Our data show that the LICA, which is easy to automate, is a promising technique for quantification of E2 in human serum and could be used for clinical detection.

## 1 INTRODUCTION

Estradiol (E2), an important and major biologically active estrogen in nonpregnant women, is a steroid hormone with a molecular mass of 272.3 Da. It is primarily produced in developmental follicles or the corpus luteum and synthesized by follicular cells and granulosa cells under the effects of follicle‐stimulating hormone and luteinizing hormone.^1^, ^2^ E2 is secreted at varying rates during the menstrual cycle throughout the period of ovarian activity. The normal level of E2 is <40 pg/mL for males. For females, the normal levels are <20 pg/mL for prepubertal children, 20‐300 pg/mL for adolescent girls, 30‐800 pg/mL during the menstrual cycle, and up to 20 000 pg/mL during pregnancy.^3^ E2 plays an indispensable role in development of the reproductive organs and secondary sexual characteristics.^4^, ^5^ Besides, measurement of serum E2 is of great value in the assessment of many diseases, including delayed sexual development or precocious puberty, abnormal menstrual cycles, menopause, ovulation induction, infertility, ectopic pregnancy, and gynecomastia.^6^, ^7^, ^8^, ^9^

Several approaches have been reported for E2 detection and measurement. Firstly, chromatographic methods include the following: high‐performance liquid chromatography,^10^ liquid chromatography‐mass spectrometry,^11^, ^12^, ^13^, ^14^, ^15^ and gas chromatography‐mass spectrometry.^16^, ^17^, ^18^ They are not accessible in all laboratories for routine analysis, because these methods require complex instruments, have high detection cost, and use complex and time‐consuming sample preparation methods. Secondly, immunological methods include the following: chemiluminescent immunoassays^19^ and electrochemical immunoassay analysis.^20^ Immunological methods are highly selective and easy to perform, but the cumbersome and tedious washing process limits their applications to some extent. Thus, it is necessary to establish a homogeneous method with no washing requirements and faster kinetics for the detection of E2.

**Figure 1:**
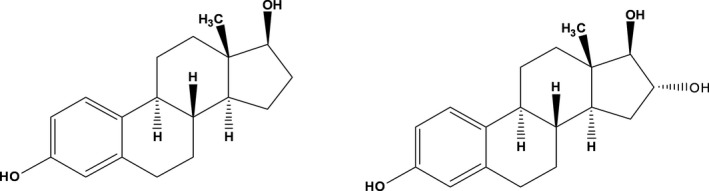
The chemical structure of estradiol (the left) and estriol (the right)

In this study, we developed a novel homogeneous light‐initiated chemiluminescent assay (LICA).^21^, ^22^, ^23^ This system uses donor and acceptor beads, which are brought into close proximity by interaction of labeled biomolecules. When the distance is within 200 nm, singlet oxygen will transfer from the donor beads to the acceptor beads under excitation, which will cause the acceptor beads to fluoresce at 520‐618 nm. This is a homogeneous method that is sensitive, specific, stable, and free of separation and washing steps and has high throughput.^24^, ^25^, ^26^, ^27^ Because the concentration of E2 varies greatly in different periods, the detection method requires a large detection interval to meet the clinical needs. Besides, E2 is a small molecule, so we chose a competitive method to achieve accurate quantification of E2 at different concentrations. For the reason, the choice of competitive antigen is crucial of this experiment. In subsequent studies, we found that using biotinylated E3 as competitive antigen, which has slightly reduced affinity toward the monoclonal anti‐human E2 antibodies compared with biotinylated E2, can significantly improve the detection sensitivity. Therefore, we chose biotinylated E3 as competitive antigen in subsequent experiments, which is an innovation of this study. E3 is a structural analog of E2 (Figure 1). In the assay described here, E2 competes with biotinylated E3 for binding to monoclonal anti‐human E2 antibodies. Donor beads coated with streptavidin are then used to capture the biotinylated E3, which brings the two bead types into close proximity. As this assay used competition between the labeled tracer and the analyte, an increase in analyte concentration will cause a signal decrease. The expected detection range of E2 is 20‐5000 pg/mL. We optimized the detection conditions and evaluated the analytical performance to establish a homogeneous chemiluminescent method for E2 detection.

## 2 MATERIALS AND METHODS

### 2.1 Samples

Clinical serum samples (n = 133) were collected from the Tianjin Central Hospital of Obstetrics and Gynecology. The samples were obtained from 106 Chinese healthy women and 27 cancer patients (ovarian cancer). The average age of them is 39 (18‐65) years old. All serum samples were stored in aliquots at −20°C.

### 2.2 Apparatus and chemicals

Acceptor beads, monoclonal anti‐human E2 antibodies, Sulfo‐NHS‐LC‐Biotin, and donor beads coated with streptavidin were purchased from Beyond Biotech. BSA‐E2 and BSA‐E3 were purchased from Abbexa. E2 for use as a calibrator was purchased from RayBiotech. Dimethyl sulfoxide, sodium borohydride, glycine, HEPES, bovine serum albumin, ProClin‐300, citric acid, sodium citrate, and sodium hydroxide were purchased from Sigma‐Aldrich AB. Measurements were performed at 37°C using a high‐throughput chemiluminescence analyzer instrument (Beyond Biotech) equipped with a 680 nm excitation source and 610 nm emission filter.

### 2.3 Biotinylation of E2 and E3

E2/E3 was linked to BSA via 6‐(O‐(carboxymethyl)oxime as a linker at position 6, and we have already purchased it. Then, E2–BSA and E3–BSA were biotinylated by Sulfo‐NHS‐LC‐Biotin dissolved in dimethyl sulfoxide and then dialyzed in 0.01 mol/L phosphate‐buffered saline overnight at 4°C. Biotin solution was added at a biotin to antigen molar ratio of 20:1, and the mixture was stirred overnight at 4°C. To remove the unbound biotin, the mixture was then dialyzed in phosphate buffer at 4°C for 24 hours (MWCO 3.5 kDa). The final product was stored at −20°C.

### 2.4 Coupling of antibody to beads

After washing twice with 200 µL of ultrapure water, acceptor beads (2 mg) were resuspended in 0.05 mol/L carbonate buffer (pH 9.6) and sonicated for 30 seconds. Monoclonal anti‐human E2 antibodies (0.1 mg) were added to the solution, and it was incubated overnight at 37°C with stirring. Then, 10 µL of reducing agent (NaBH4, 8 mg/mL) was added, and the mixture was incubated for 2 hours at room temperature. Next, blank binding sites were blocked with 40 µL of glycine (75 mg/mL) for 1 hour at room temperature. After repeated washing with ultrapure water, the mixture was resuspended in 200 µL of a preservation solution, which consisted of 0.02 mol/L HEPES, 0.2% bovine serum albumin, and 0.1% Proclin‐300.

### 2.5 Assay procedure

**Figure 2:**
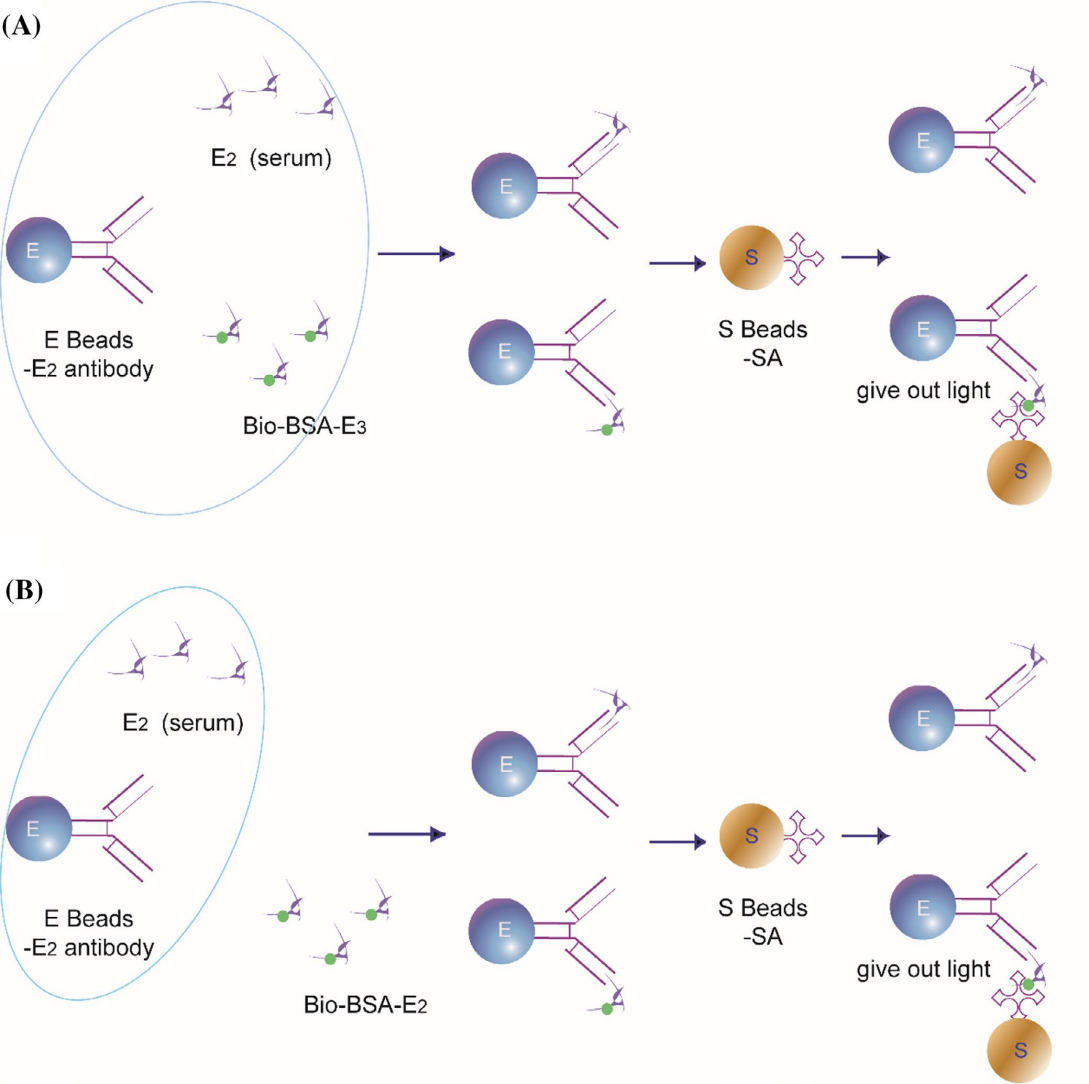
Schematic diagram of LICA. A, For the one‐step method. B, For the two‐step method

The theory of LICA for quantitation of E2 is illustrated in Figure 2. In this study, we compared one‐step and two‐step methods using biotinylated E2 as a competitive antigen. We also compared the one‐step method using biotinylated E3 as a competitive antigen and the two‐step method using biotinylated E2. The release buffer was 0.01 mol/L citric acid‐sodium citrate buffer (pH 4.0), the same as antigen diluent. The antibody diluent was 0.005 mol/L glycine‐sodium hydroxide buffer (pH 8.6). In the one‐step method, 25 µL of release agent, 25 µL of serum sample or calibrators, 25 µL of biotin‐BSA‐E2/E3 conjugate, and 25 µL of monoclonal anti‐human E2 antibodies were added sequentially to the microplate, which was then placed in the chemiluminescence analyzer for 17 minutes at 37°C. Then, 175 µL of donor beads coated with streptavidin was added to the plate, which was placed under green light in the analyzer for another 15 minutes. The light signal was measured by the instrument. Samples were analyzed in duplicate. In the two‐step method, serum samples or calibrators were incubated with antibodies first, and then, biotin‐labeled reagents were added to react with the remaining antibodies.

### 2.6 Calibration

The E2 calibrators were diluted with 0.01 mol/L citric acid‐sodium citrate buffer (pH 4.0) to concentrations of 0, 100, 600, 1200, 2400, and 4800 pg/mL. A four‐parameter logistic model was used to construct the standard curve, which had an inverse correlation between the concentration of E2 calibrators and the signal.

### 2.7 Optimization of the reaction criteria

Many factors could influence the reaction criteria. We optimized the competition mode, biotinylated antigens, concentration of biotinylated antigens, and monoclonal anti‐human E2 antibodies.

### 2.8 Assay performance characteristics of E2 measurement by LICA

#### 2.8.1 Sensitivity

Firstly, we measured the zero calibrators for 15 times and then calculated the mean and the standard deviation (SD) of the signal. The analytical sensitivity was calculated using mean + 2SD by the standard curve. Samples with low concentrations were diluted, and each diluted sample was tested 20 times on separate days. The maximum dilution concentration at 20% of coefficient of variation (CV) was selected as the functional sensitivity of the detection system.

#### 2.8.2 Precision

The precision was evaluated by performing duplicate tests with E2 at high and low concentrations. Two quality control serum samples of E2 were selected. The intra‐assay precision was determined using 10 consecutive measurements for two serum pools in the same analysis test. To determine the inter‐assay variation, two spiked samples were analyzed using the same protocol on 30 consecutive days.

#### 2.8.3 Recovery test

Serum samples with high and low concentrations of E2 were selected, which were added equal volume of serum samples with different concentrations of E2, respectively. The concentrations of the samples before and after addition were detected, and then, the recovery rates were calculated.

#### 2.8.4 Interference experiment

Interference was evaluated with three serum pools by adding bilirubin, hemoglobin, and triglyceride at different concentrations. The interference rates for E2 in these samples were calculated from the concentrations of E2 determined before and after addition of the interfering substances.

#### 2.8.5 Cross‐reactivity

To evaluate the specificity of the LICA assay, different concentrations of structural analogs and some reproductive hormones were added into serum samples with different concentrations of E2. The cross‐reactivity was calculated from E2 concentrations determined before and after the addition of analogs.

#### 2.8.6 Comparison with clinical instruments

The LICA system was compared with two clinical methods for detecting E2: the IMMULITE 2000 System and the VIDAS System. Serum samples from 133 clinical patients were chosen for this experiment. Regression analysis was conducted to evaluate the correlation between the LICA system and the two methods.

## 3 RESULTS

### 3.1 Calibration curve

**Figure 3:**
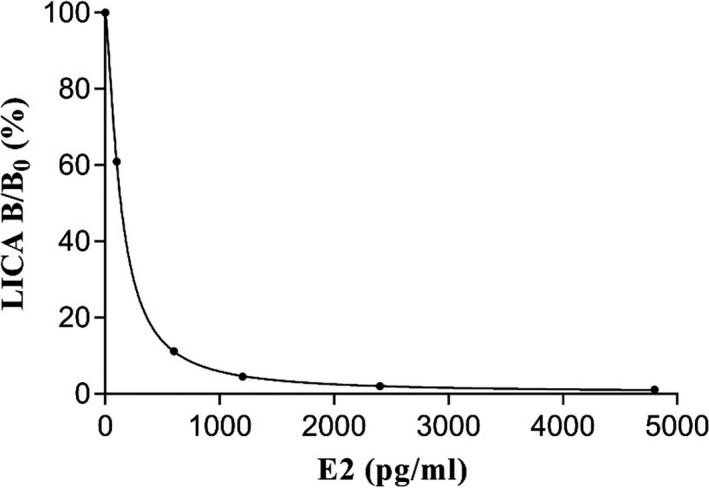
Calibration curve of the LICA for detecting E2 in human serum. The error bars were invisible because they were shorter than the height of the symbol

A standard dose‐response curve (*y* = 99.59/[1 + (*x*/136.03)^1.42^] + 0.40) was created with six concentrations of calibrators, including zero reference calibrators. The correlation coefficient was .999. The calibration curve is shown in Figure 3.

### 3.2 Optimization of competition mode

**Figure 4:**
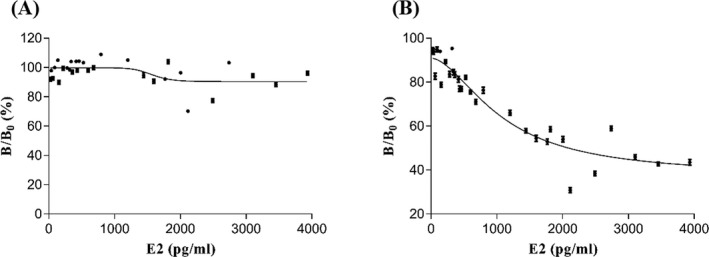
Optimization of competition mode. A, One‐step method. B, Two‐step method

In the one‐step equilibrium assay, the serum samples, monoclonal anti‐human E2 antibodies, and biotinylated E2 antigen were added simultaneously. After incubation at 37°C for 17 minutes, the LICA Common Reagent containing the SA‐S‐beads was added before another 17‐minutes incubation. Finally, the signal was recorded. In the two‐step sequential assay, serum samples and monoclonal anti‐human E2 antibodies were added to the microplate first. Then, biotinylated E2 antigen was added after incubation at 37°C for 17 minutes. This was followed by another 17‐minutes incubation, and the LICA Common Reagent containing the SA‐S‐beads was added. Finally, the signal was measured using the LICA instrument. The above two methods were used to test 30 serum samples, and a dose‐response curve for E2 was established. The fitting coefficients were 0.2494 and 0.8908 for the one‐step and two‐step methods, respectively (Figure 4). These results show that the two‐step method is superior to the one‐step method when using biotinylated E2 antigen. However, samples with low concentrations could not be distinguished well because of the stronger binding ability between biotinylated E2 antigen and anti‐human E2 antibodies than E2 in serum samples. The analytical sensitivity was 75 pg/mL when we used biotinylated E2 antigen by two‐step method. The result indicated that low concentrations of E2 cannot be detected. Thus, we considered replacing the biotinylated E2 antigen with its structural analog. It had lower affinity to anti‐human E2 antibodies.

### 3.3 Optimization of biotinylated antigens

**Figure 5:**
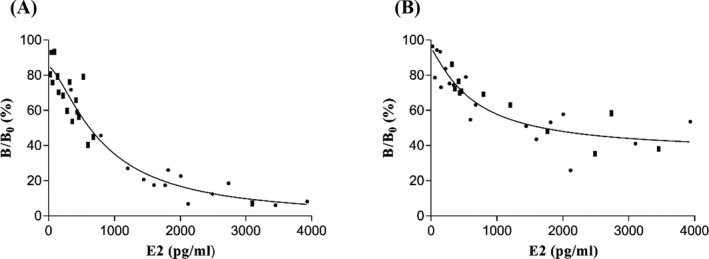
Optimization of biotinylated antigen. A, One‐step method with biotinylated E3 antigen. B, Two‐step method with biotinylated E2 antigen

Thirty serum samples were tested using the one‐step method with the structural analog (biotinylated E3 antigen). The samples were tested using the two‐step method described above. To assess the assay conditions, we established a dose‐response curve for the biotinylated antigens. The fitting coefficients for the one‐step and two‐step methods were 0.9269 and 0.8081, respectively. A better fitting curve was obtained when we replaced the biotinylated E2 antigen with its structural analog (Figure 5). We also compared the CVs of a low concentration of E2 (62 pg/mL) between the two methods mentioned above in this paragraph. The CVs of intra‐assay were 8.11% and 16.82% for the one‐step and two‐step methods, respectively, while the CVs of inter‐assay were 10.35% and 18.61%, respectively. The results showed that an analyte competitor with a slightly reduced affinity toward the antibody is peculiarly important, by which the method would have a better precision and sensitivity. The one‐step method is easier to operate and time‐saving than the two‐step method. Therefore, we selected the biotinylated E3 antigen and one‐step equilibrium assay for subsequent experiments.

### 3.4 Optimization of the concentration of monoclonal anti‐human E2 antibodies and biotinylated E3 antigen

**Figure 6:**
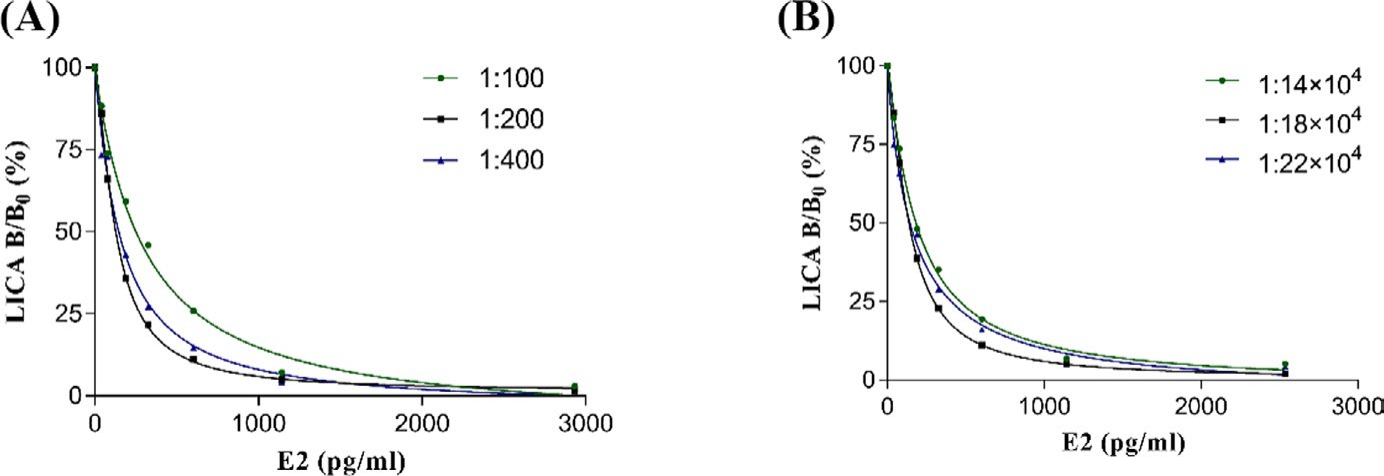
Optimization of LICA‐E2 reacting conditions. A, Optimization of the concentration of monoclonal anti‐human E2 antibodies. B, Optimization of the concentration of biotinylated E3

Nine samples were selected with concentrations ranging from 0 to 3000 pg/mL. According to our prior knowledge, we performed 1:100, 1:200, and 1:400 dilutions on the monoclonal anti‐human E2 antibodies and serially diluted the biotinylated E3 antigen at 1:14 × 10^4^, 1:18 × 10^4^, and 1:22 × 10^4^ in assay buffer. We selected a serum sample with a low concentration of E2 (84 pg/mL) to evaluate the CV. The CVs were 33.98%, 15.68%, and 44.24% for the 1:14 × 10^4^, 1:18 × 10^4^, and 1:22 × 10^4^ dilutions, respectively. The 1:18 × 10^4^ dilution of biotinylated E3 antigen had better precision than the other dilutions we investigated for serum samples with low concentrations. When the antibody was diluted to 1:200, a maximum slope was obtained (Figure 6). Therefore, dilutions of 1:200 and 1:18 × 10^4^ were used in subsequent experiments.

### 3.5 Assay performance

#### 3.5.1 Assay sensitivity

**Figure 7:**
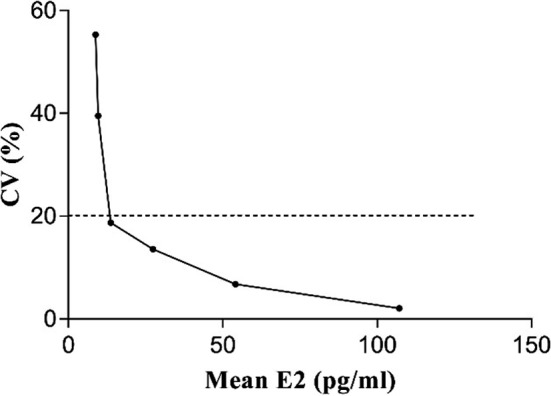
Functional sensitivity of E2 by LICA

The mean and standard deviation of the signal value were 158 440 and 2425.80, respectively. According to the standard curve, the analytical sensitivity was 7.16 pg/mL. Sera with low concentrations (106.7 pg/mL) were selected for a series of dilutions, and 20 repeated tests were performed for each diluted sample. The average concentrations for all the diluted samples were 107.2, 54.1, 27.4, 13.7, 9.6, and 8.8 pg/mL. The functional sensitivity of the detection system was determined as the 20% between‐run CV, as recommended by the National Academy Clinical Biochemistry.^28^ When the average concentration was 13.7 pg/mL, the corresponding CV was 18.71% (Figure 7). The functional sensitivity was approximately 13.7 pg/mL.

#### 3.5.2 Assay precision

The assay was performed with calibrators and samples in duplicate (Table 1). In the intra‐assay study, the CV ranged from 3.16% to 8.02%. In the inter‐assay for E2, the CV ranged from 3.30% to 10.26%. The results showed remarkable repeatability, which could be attributed to lack of washing steps, automation of the method, and rapid analysis. In conclusion, the LICA provided good results for E2 intra‐ and inter‐assay variability.

**Table 1:** The intra‐assay precision and inter‐assay precision

Group	Intra‐assay (n = 10)			Inter‐assay (n = 30)		
	Mean	s	CV (%)	Mean	s	CV (%)
Low	31.8	2.6	8.02	34.4	4.2	10.26
High	198.5	6.3	3.16	197.0	6.5	3.30

#### 3.5.3 Recovery test

Concentrations of 78.5 and 145.1 pg/mL were selected for adding into serum samples with low (121.3 pg/mL) and high (2713.2 pg/mL) concentrations. The recovery rates ranged from 97.5% to 106.8% as shown in Table 2 and indicated that the assay could be used for precise quantification of E2 in clinical samples.

**Table 2:** The recovery test of LICA

Group	Added E2 (pg/mL)	Expected E2 (pg/mL)	Measured E2 (pg/mL)	Recovery (%)
Low value	78.5	199.8	196.6	98.4
	145.1	266.4	275.4	103.4
High value	78.5	2791.7	2721.9	97.5
	145.1	2858.3	3052.5	106.8

#### 3.5.4 Interference experiment

Interference testing was implemented as described earlier in the text. E2 concentrations of 1529.6, 227.2, and 95.3 pg/mL were selected. The interference rates of the assay are shown in Table 3. The interference rate of E2 ranged from −3.6% to 5.4% and met E2 detection requirements for hyperbilirubinemia, hemolysis, and lipemia in clinical specimens.

**Table 3:** Interference from addition of hemoglobin, total bilirubin, and triglyceride to serum samples

Interfering substance	High	Interference (%)	Middle	Interference (%)	Low	Interference (%)
Bilirubin (mg/mL)						
0	1529.6		227.2		95.3	
20	1446.3	5.4	235.3	−3.6	90.6	4.9
Hemoglobin (mg/dL)						
0	1529.6		227.2		95.3	
500	1565.7	−2.4	220.1	3.1	100.2	−5.1
Triglyceride (mg/dL)						
0	1529.6		227.2		95.3	
500	1473.4	3.7	231.9	−2.1	91.1	4.4

#### 3.5.5 Specificity

Cross‐reactivity was assessed by adding known concentrations of estriol (20 ng/mL), ethinyl‐estradiol (20 ng/mL), and testosterone (10 000 ng/mL) into definite concentrations of E2 (high, 1360.4 pg/mL; middle, 242.5 pg/mL; low, 75.3 pg/mL). As shown in Table 4, the ratio of cross‐reactivity ranged from 1.9% to 10.6% which indicated that no major cross‐reaction was identified between E2 and structural analogs. At the meantime, the influence by some reproductive hormones could also be ignored.

**Table 4:** Specificity test with structural analogs to estradiol

Steroid	High	Cross‐reactivity (%)	Middle	Cross‐reactivity (%)	Low	Cross‐reactivity (%)
Estriol (ng/mL)						
0	1360.4		242.5		75.3	
20	1388.6	2.0	255.8	5.2	84.2	10.6
Ethinyl‐estradiol (ng/mL)						
0	1360.4		242.5		75.3	
20	1392.8	2.3	256.9	5.6	84.1	10.5
Testosterone (ng/mL)						
0	1360.4		242.5		75.3	
10 000	1386.4	1.9	251.6	3.6	83.8	10.1

#### 3.5.6 Comparison with clinical instruments

**Figure 8:**
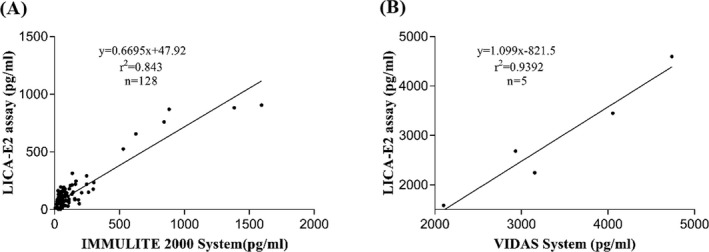
Correlation between E2 values in serum samples measured by LICA and clinical instruments. A, Comparison with the IMMULITE 2000 System. B, Comparison with the VIDAS System

Majority of the samples (n = 128) were analyzed by the IMMULITE 2000 System and the others were analyzed by the E2 VIDAS System, since the reportable range of the IMMULITE 2000 System is 15‐2000 pg/mL. The samples were also tested by the LICA. The results from the LICA analyzer showed high accordance with those from the other two systems. The correlation coefficients were 0.843 and 0.939 with the IMMULITE 2000 and VIDAS systems, respectively (Figure 8). This comparison confirmed the accuracy of the LICA system.

## 4 DISCUSSION

The essential principle of LICA is energy transfer of singlet oxygen atoms in the luminescent oxygen channeling immunoassay (LOCI). LOCI is a homogeneous immunoassay method, which can provide rapid analysis of small molecules in serum samples.^21^ The influence of steric hindrance on the hapten can be reduced by biotinylation.^29^ In this study, we established a novel assay based on LOCI using E3 instead of E2 as a competitive antigen for quantification of E2 in human serum.

The LICA system is a microplate‐based homogeneous chemiluminescent immunoassay that relies on a competitive assay design. E2 in serum samples competes with biotinylated antigen for the same binding site on the anti‐human E2 antibodies which is in limited amounts. Given that the two‐step method is cumbersome and time‐consuming, we selected the one‐step method. In view of the poor fitting coefficient of the one‐step method and poor discrimination of low concentration samples of the two‐step method, we made some changes to the competitive antigen, which plays a key role in this method. When using biotin‐BSA‐E2 as the competitive antigen, we could not distinguish low concentrations of E2 in serum, which might be attributed to the strong binding force between biotin‐BSA‐E2 and anti‐human E2 antibodies. Because the antibody affinities of the competitor and the analyte strongly influence the sensitivity, equilibrium has to be reached. Consequently, an analyte competitor with reduced affinity toward the antibody is able to shift the equilibrium in favor of the sample analyte, which is especially important for low analyte concentrations.^30^, ^31^ Therefore, we chose biotin‐BSA‐E3 as the competitive antigen. E3 is a metabolite of E2 and estrone, which is less active than E2. E3 and E2 have very similar structures, and the only difference is that there is one more hydrogen atom on the 16th carbon atom in E3 than in E2. The results indicated that the low‐affinity competitor greatly improved the analytical sensitivity of the LICA. Accordingly, biotinylated E3 was chosen to perform the LICA.

In this competitive immunoassay, E2 and biotinylated E3 compete for a limited number of antibody binding sites. Therefore, the concentration of competitor and detection antibody may affect the performance of the immunoassay.^32^ The sensitivity of this assay is highly dependent on the biotin‐BSA‐E3 concentration. We performed serial dilutions of the monoclonal anti‐human E2 antibody at 1:100, 1:200, and 1:400 in assay buffer to obtain a better fitting coefficient and detection sensitivity. The detection range of method should meet clinical demands. It was affected by the concentration of the detection antibody, which was determined by the analyte and labeled antigen concentration. To optimize the conditions of this assay, we performed serial dilutions of the biotinylated E3 antigen at 1:14 × 10^4^, 1:18 × 10^4^, and 1:22 × 10^4^ in assay buffer to obtain a wider detection range. The optimum dilutions for the monoclonal anti‐human E2 antibody and biotinylated E3 antigen were 1:200 and 1:18 × 10^4^, respectively.

The equilibrium saturation E2‐LICA is a rapid and sensitive assay that does not require the tedious washing steps of other methods.^33^, ^34^, ^35^, ^36^ The analytical sensitivity of the E2‐LICA was 7.16 pg/mL, and greater than the sensitivities of other methods.^19^ Each of the sensitizer beads generates 60 000 singlet oxygen atoms under excitation, which leads to the high sensitivity of the method. The intra‐ and inter‐assay precision both showed remarkable repeatability, which could be attributed to the lack of washing requirements and the automatic procedure. The recovery rates ranged from 97.5% to 106.8%, which indicated the method had high specificity. Moreover, the assay was not affected by the interferents such as bilirubin, hemoglobin, and triglyceride. In addition, the cross‐reactivity results varied from 1.9% to 10.6%, which showed that LICA assay is highly specific for E2. The IMMULITE 2000 System is an enzyme‐labeled chemiluminescent competitive immunoassay,^22^ which has a narrow detection range and is unable to detect high E2 concentrations in clinical serum samples. The VIDAS System combines an enzyme immunoassay and fluorescent assay^37^, ^38^ and uses an expensive reagent and requires manual operation. Both of these methods are very time‐consuming. By comparison, the reagents used in the LICA system are readily available and inexpensive, which means this system will be easy to realize commercially and automate.

In summary, our data show that the LICA is a promising method for the quantification of E2 in human serum. LICA achieves homogeneous and simple detection with its unique technology. Compared with traditional immunoassay methods, LICA has superior detection performance and shows promise as an important tool for clinical detection.

## CONFLICT OF INTEREST

The authors declare no conflicts of interest.

## ETHICAL APPROVAL

The work was supported by the Ethics Committee of Tianjin Medical University (TMUHMEC2017008) and conducted in accordance with the principles of the Declaration of Helsinki. The sera involved in our research were from clinical patients. Informed consent was obtained from all human participants.
